# CPT1C-mediated fatty acid oxidation facilitates colorectal cancer cell proliferation and metastasis: CPT1C promotes CRC progression

**DOI:** 10.3724/abbs.2023041

**Published:** 2023-04-20

**Authors:** Jing Li, Wanwei Zheng, Jie Wu, Jun Zhang, Bin Lv, Wenshuai Li, Jie Liu, Xin Zhang, Tiansheng Huang, Zhongguang Luo

**Affiliations:** 1 Department of CyberKnife Center Huashan Hospital Fudan University Shanghai 200040 China; 2 Department of Digestive Diseases of Huashan Hospital Fudan University Shanghai 200040 China; 3 Department of Oncology the First Affiliated Hospital of Soochow University Suzhou 215000 China; 4 Institute of Translational Medicine Shanghai Jiaotong University Shanghai 200025 China; 5 Department of Digestive Diseases Shanghai Guanghua Hospital of Integrated Traditional Chinese and Western Medicine Shanghai University of Traditional Chinese Medicine Shanghai 200052 China

**Keywords:** colorectal cancer, fatty acid oxidation, CPT1C, cell proliferation, cell migration

## Abstract

Fatty acid oxidation (FAO) has been proven to be an accomplice in tumor progression. Carnitine palmitoyltransferase 1C (CPT1C), a rate-limiting enzyme in FAO, mainly functions to catalyze fatty acid carnitinylation and guarantee subsequent entry into the mitochondria for FAO in colorectal cancer (CRC). Gene expression data and clinical information extracted from The Cancer Genome Atlas (TCGA) database show significantly higher expression of CPT1C in patients with metastatic CRC ( *P*=0.005). Moreover, overexpression of CPT1C is correlated with worse relapse-free survival in CRC (HR 2.1, *P*=0.0006), while no statistical significance is indicated for CPT1A and CPT1B. Further experiments demonstrate that downregulation of CPT1C expression leads to a decrease in the FAO rate, suppression of cell proliferation, cell cycle arrest and repression of cell migration in CRC, whereas opposite results are obtained when CPT1C is overexpressed. Furthermore, an FAO inhibitor almost completely reverses the enhanced cell proliferation and migration induced by CPT1C overexpression. In addition, analysis of TCGA data illustrates a positive association between CPT1C expression and HIF1α level, suggesting that CPT1C is a transcriptional target of HIF1α. In conclusion, CPT1C overexpression indicates poor relapse-free survival of patients with CRC, and CPT1C is transcriptionally activated by HIF1α, thereby promoting the proliferation and migration of CRC cells.

## Introduction

Colorectal cancer (CRC) is one of the most common cancers worldwide [1]. Radical operation followed by adjuvant chemotherapy has been well recognized as a treatment standard for resectable CRC, yet the prognosis of advanced CRC remains unsatisfactory [2]. Therefore, more effective prognostic markers are warranted, and the underlying molecular mechanisms for CRC progression need to be elucidated.

Metabolic reprogramming has been proven to be a critical hallmark of cancer progression [ 3– 5] . Abnormal alteration of lipid metabolism is one of the most essential metabolic characteristics of cancer cells [ 6– 8] . In response to energy stress and to maintain rapid cell proliferation, cancer cells tend to abnormally activate fatty acid oxidation (FAO) [ 9, 10] . FAO is a metabolic process catalyzed by several critical enzymes. Moreover, carnitine palmitoyltransferase 1 (CPT1) functions as one of the key rate-limiting FAO-related enzymes and transports fatty acids into the mitochondria [ 11– 13] . Previous reports have revealed that CPT1 can accelerate tumor progression in several cancer types, suggesting its potential as a promising therapeutic target [ 14– 16] . CPT1 inhibitors have been developed and have been demonstrated to suppress cell proliferation *in vitro*. Nevertheless, only a few studies have analyzed the prognostic and carcinogenic roles that CPT1 might play in CRC, and the functions of CPT1C, an isoform of CPT1, in CRC have insufficiently been explored. There are three isoforms of CPT1, including CPT1A, CPT1B and CPT1C [17].

In the present study, we found that increased expression of CPT1C is significantly linked to poor relapse-free survival (RFS) in CRC (HR 2.1, *P*=0.0006), while no statistical significance is found for CPT1A and CPT1B. Further experiments showed that CPT1C overexpression markedly enhances cell proliferation and promotes cell migration ability, whereas *CPT1C* silencing shows the opposite effects.

## Materials and Methods

### The Cancer Genome Atlas (TCGA) and GEPIA databases

mRNA expression data of CRC patients were downloaded from the TCGA database ( https://portal.gdc.cancer.gov/). Patients with RNA-seq data and complete clinical information were included for analysis. Clinicopathological parameters, such as sex, age, tumor stage, histological type, and RFS data, were extracted. The web-based tool GEPIA database ( http://gepia2.cancer-pku.cn/#analysis), with customized options, was utilized to analyze TCGA data, which could help perform differential gene expression analysis, survival analysis and gene expression correlation analysis [18]. Specifically, the GEPIA web tool was applied to analyze the expression difference of CPT1s among different tumor stages and explore the association of its expression with RFS based on the TCGA cohort. In the GEPIA analysis, the median expression level of CPT1s was set as the cut-off value for subsequent grouping.

### Cell culture

In the present study, all CRC cell lines (HCT116, DLD1, RKO and HCT8) were obtained from the National Cancer Institute (NCI, Bethesda, USA). DMEM (HyClone, Utah, USA) was supplemented with 10% fetal bovine serum (Thermo Fisher Scientific, Waltham, USA), which was then applied for the culture of all cells at 37°C with 5% CO _2_ in the incubator. Four CRC cell lines with different aggressive features were utilized in this study.

### Stable transfection

The coding sequence of the human *CPT1C* gene was amplified first and then cloned into the lentiviral vector pCDH-CMV-MCS-EF1-Puro to generate pCDH-CPT1C. The CPT1C shRNAs and scrambled shRNA were purchased from Vigene Biosciences Inc. (Rockville, USA). pLKO.1-shCPT1C#1 (shRNA1: AAAGGCATCTCTCACGTTTCTGG) and pLKO.1-shCPT1C#2 (shRNA2: AATTATGTCAGTGACTGGTGGGA) were transfected into HCT116 and RKO cells using Lipofectamine 3000 reagent (No. L3000008; Thermo Fisher Scientific, Waltham, USA) according to the manufacturer’s instructions to guarantee stable *CPT1C* knockdown (HCT116-CPT1C-NC/KD and RKO-CPT1C-NC/KD, respectively). The scrambled shRNA (GTGAGTACCGCTGCTCTACATTA) was also transfected into cells as the negative control. Meanwhile, pCDH-CPT1C and the control vector were transfected into HCT8 and DLD1 cells using Lipofectamine 3000 reagent After culture for 48 h, puromycin (Solarbio, Shanghai, China) was added to the media to screen cells with successful *CPT1C* knockdown or overexpression.

### RNA extraction and qRT-PCR

Total RNA was extracted from the aforementioned CRC cells using Trizol reagent (Invitrogen, Carlsbad, USA), after which the concentration and purity of the RNA samples were evaluated. Subsequently, it was reverse transcribed into cDNA by using PrimeScript™ RT Master Mix (Takara, Dalian, China). Finally, qRT-PCR was conducted using ABI 7900HT Real-Time PCR System (Applied Biosystems, Frederick, USA) with the following cycle conditions: 95°C for 5 s and 60°C for 30 s (40 cycles). The expression levels of related genes were calculated using QuantStudio Real-Time PCR Software. *β-actin* was used as the internal reference gene. The primers used in this study were as follows: *β-actin* forward 5′-CATGTACGTTGCTATCCAGGC-3′, reverse 5′-CTCCTTAATGTCACGCACGAT-3′ and *CPT1C* forward 5′-ATGGGAATGCGCCCCTTATG-3′, reverse 5′-AGGTGGCGGATGTAGTCTTTT-3′.

### Cell viability and cell cycle assays

The viability of CRC cells was examined using a CCK-8 assay kit (DOJINDO, Tokyo, Japan), and the cell cycle distribution of CRC cells was tested using a cell cycle kit (BD, Franklin Lakes, USA) according to the manufacturer’s guidelines. Briefly, the procedure of CCK-8 assay involves seeding cells in a 96-well plate and then adding CCK-8 reagent to the plate. After incubation for 2 h, we measure the absorbance of the wells at 450 nm using a microplate reader. Lastly, we calculate the relative cell viability of the cells by comparing the absorbance of experimental wells with control wells containing untreated cells. The procedure of cell cycle test involves fixing cells with ethanol, staining them with fluorescent dyes that bind to DNA, and then analyzing the stained cells using flow cytometry. The flow cytometer measures the intensity of the fluorescence emitted by each cell, which reflects the amount of DNA that is present in the cell.

### Migration assay

For transwell assays, 2×10 ^4^‒4×10 ^4^ CRC cells were seeded into the upper chamber of transwell (Corning, New York, USA) without FBS supplementation, while 500 μL DMEM with 10% FBS was added into the lower chamber. After 48 h of culture, migrated cells were fixed with 4% paraformaldehyde, and then stained with crystal violet staining solution. Finally, the number of migrated cells were counted under a microscope.

### FAO assay

The FAO rate was evaluated using an FAO assay kit (ab222944; Abcam, Cambridge, UK) according to the manufacturer’s instructions. Briefly, cells were plated in a 96-well black plate with a clear bottom at a density of 2×10 ^4^ cells/well. Subsequently, cells were washed twice with measurement media devoid of fatty acids (FA) (comprising 0.5 mM L-carnitine and 2.5 mM glucose in the base measurement media). Post-washing, 90 μL of FA measurement media (incorporating 150 μM oleate-BSA conjugate) and 10 μL of extracellular O _2_ consumption reagent were added. Each well was then sealed with 100 μL of high sensitivity mineral oil and fluorescence measurements were read every 1.5 min for 60 min at Ex/Em of 360/670 using the Spark™ 10M multimode microplate reader (Tecan, Beijing, China).

### Western blot analysis

RIPA buffer (Thermo Fisher Scientific) was applied to lyze CRC cells and the total protein in the lysates were then quantified using a BCA Protein Assay kit (Thermo Fisher Scientific). Cellular proteins were resolved by sodium dodecyl sulfate polyacrylamide gel electrophoresis (SDS-PAGE). Afterwards, proteins were transferred onto polyvinylidene difluoride membranes. Subsequently, after being blocked, membranes were incubated with primary antibodies against β-actin (1:1000 dilution; Abcam), HIF1α (1:1,000 dilution; Proteintech, Wuhan, China) and CPT1C (1:1000 dilution; Proteintech) overnight at 4°C, followed by incubation with the corresponding HRP-conjugated secondary antibody. Finally, ECL reagents (Thermo Fisher Scientific) were used for blot visualization and quantification on a BioImaging System (Tanon 5200, Shanghai, China). β-actin was used as the loading control.

### Luciferase reporter assay

The promoter region of *CPT1C* was cloned into GL3-basic, the luciferase reporter vector. DLD1 and HCT8 cells were cultured, followed by cotransfection with pRL-TK vectors, pGL3-basic-CPT1C, and HIF1α overexpression plasmids. After transfection for 48 h, a Dual-Luciferase Reporter Assay System (Promega, Madison, USA) was used to evaluate the luciferase reporter activity according to the manufacturer’s instructions.

### Chromatin immunoprecipitation (ChIP) assay

The HIF1α-binding site on the *CPT1C* promoter was identified by using the online tool JASPAR ( https://jaspar.genereg.net/). The binding status of HIF1α on the promoter region of *CPT1C* was assessed using a chromatin immunoprecipitation kit (Cell Signalling Technology, Beverly, USA) according to the standard manuals. Briefly, to stimulate cross-linking, cells were exposed to 1% formaldehyde for 10 min at 37°C. Following this, glycine was utilized to terminate formaldehyde fixation post-cross-linking. After PBS washing, cells were resuspended in 300 μL of lysis buffer containing 1 mM PMSF, 1% SDS, 10 mM EDTA, and 50 mM Tris (pH 8.1) to produce fragmented DNA via sonication. Supernatant was cleared using a mixture of protein G-Sepharose and herring sperm DNA. Subsequently, the recovered supernatant was subjected to a 2-h incubation with specific antibodies or isotype control IgG, with protein G-Sepharose beads and herring sperm DNA, followed by antibody denaturation via 1% SDS in lysis buffer. Precipitated DNA underwent extraction from beads via immersion in 1.1 M NaHCO _3_ solution and 1% SDS solution at 65°C for 6 h, and immunoprecipitated DNA was retrieved from the beads using a similar method. Purification of DNA was conducted as the last step.

### Statistical analyses

R software (R version 3.2.5; https://www.r-project.org/) was utilized for all statistical analyses. Data are shown as the mean±standard deviation (SD). Student’s *t* test was conducted for data with a normal distribution; while for data with a skewed distribution, the Wilcoxon rank-sum test was conducted to compare the differences between different groups. Patients were divided into high and low groups based on the expression levels of CPT1. RFS was calculated by the Kaplan-Meier method, and the survival differences between the low and high CPT1 expression groups were compared by the log-rank test. *P*<0.05 was considered statistically significant.

## Results

### High expression of CPT1C is linked to advanced tumor stage and increased relapse risk in CRC

**Figure 1:**
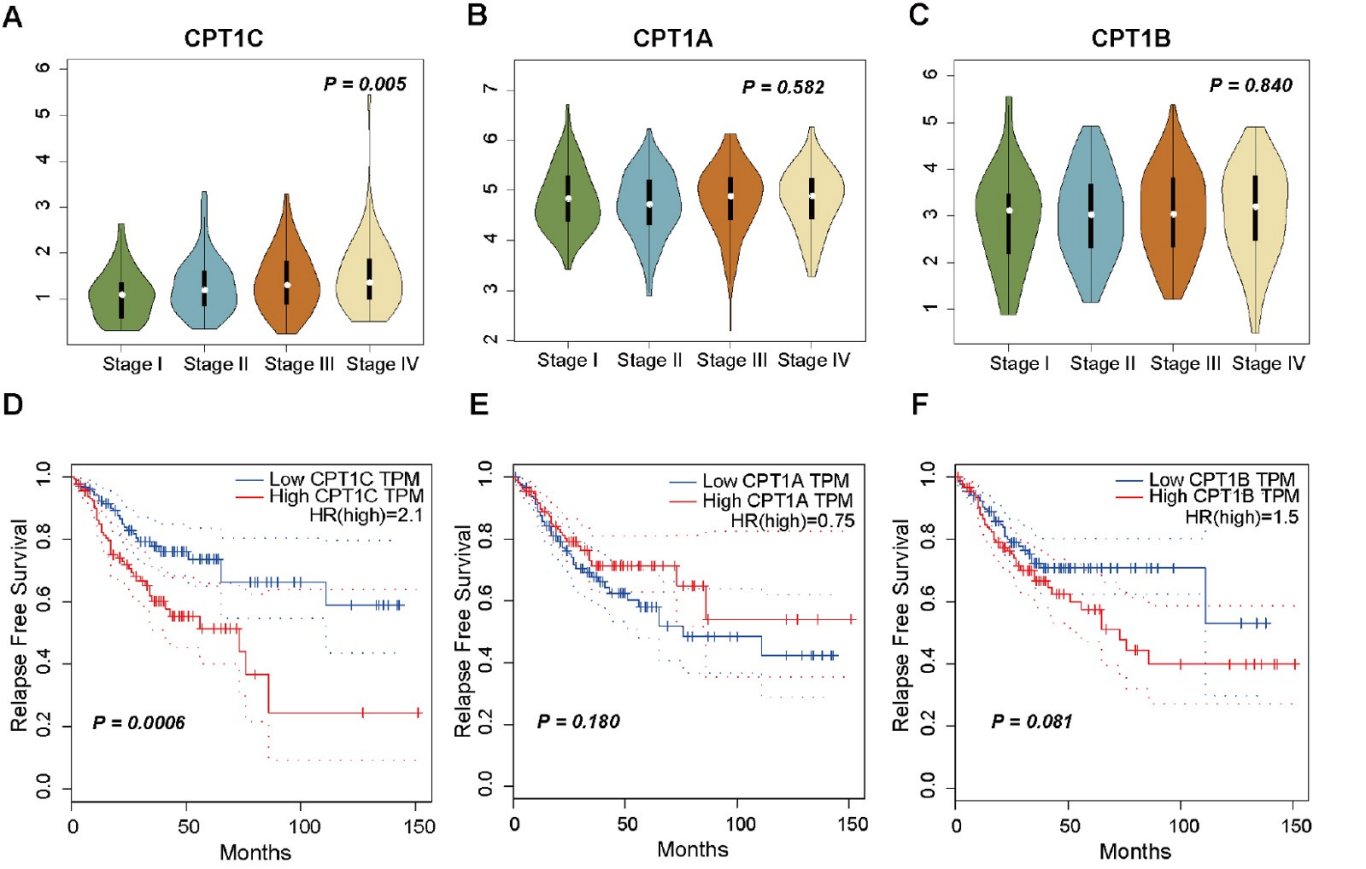
Figure 1 Distribution and survival analysis of CPT1s in TCGA CRC patients (A‒C) Distribution of CPT1C, CPT1A and CPT1B expressions among different tumor stages in CRC patients from the TCGA cohort. (D‒F) Kaplan-Meier survival curves of RFS comparing low and high expressions of CPT1C (D), CPT1A (E) and CPT1B (F). The P value in (A‒C) was calculated using one-way ANOVA followed by Dunnett’s multiple comparisons. The P value in (D‒F) was calculated using the log-rank test.

The GEPIA web tool was used to depict the expression levels of CPT1s, including CPT1A, CPT1B and CPT1C, among different tumor stages in the TCGA cohort to evaluate the correlation between tumor stage and CPT1s expression. Notably, the expression level of CPT1C is positively associated with tumor stage, and metastatic patients (stage IV) show notably higher expression of CPT1C ( *P*=0.005; Figure 1A), indicating the potential role of CPT1C in promoting CRC metastasis. However, no significant difference was noticed for CPT1A ( *P*=0.582; Figure 1B) and CPT1B ( *P*=0.840; Figure 1C) expressions among different tumor stages. Furthermore, we compared the expression of CPT1C between 64 paired cancer and adjacent normal tissues from our cancer center and found that CPT1C is highly expressed in cancer tissues ( Supplementary Figure S1A).

To further explore the correlation between CPT1s expression and CRC relapse, we divided patients from TCGA into low and high expression level groups using the median value of CPT1s expression levels as the cut-off. The Kaplan-Meier estimator demonstrated that overexpression of CPT1C is significantly associated with poor RFS (HR 2.1; *P*=0.0006; Figure 1B). Nevertheless, the expression levels of CPT1A (HR 0.75; *P*=0.180; Figure 1C) and CPT1B (HR 1.5; *P*=0.081; Figure 1D) statistically show no correlation with patient survival.

### CPT1C promotes CRC cell proliferation and migration

**Figure 2:**
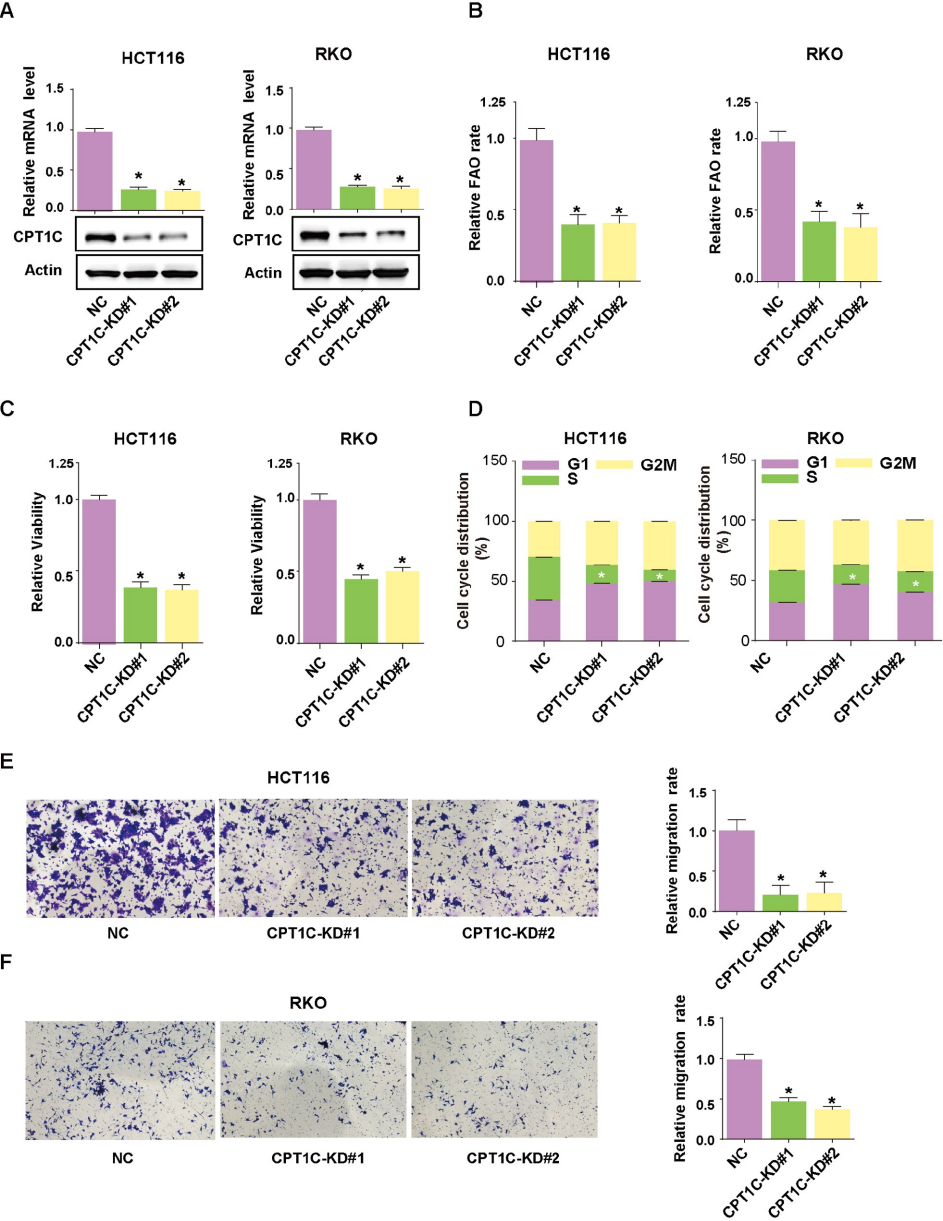
Figure 2 Silencing of *CPT1C* inhibits CRC cell proliferation and migration (A) qRT-PCR and western blot analysis showing short hairpin RNA-mediated deletion of CPT1C. (B,C) CCK-8 assay showing the viability of HCT116 (B) and RKO cells (C) at the indicated time points. (D) CPT1C silencing induced cell cycle arrest. (E,F) CPT1C silencing suppressed cell migration ability. Data are presented as the mean±SD. The P value was calculated using one-way ANOVA followed by Dunnett’s multiple comparisons. * P<0.05.

**Figure 3:**
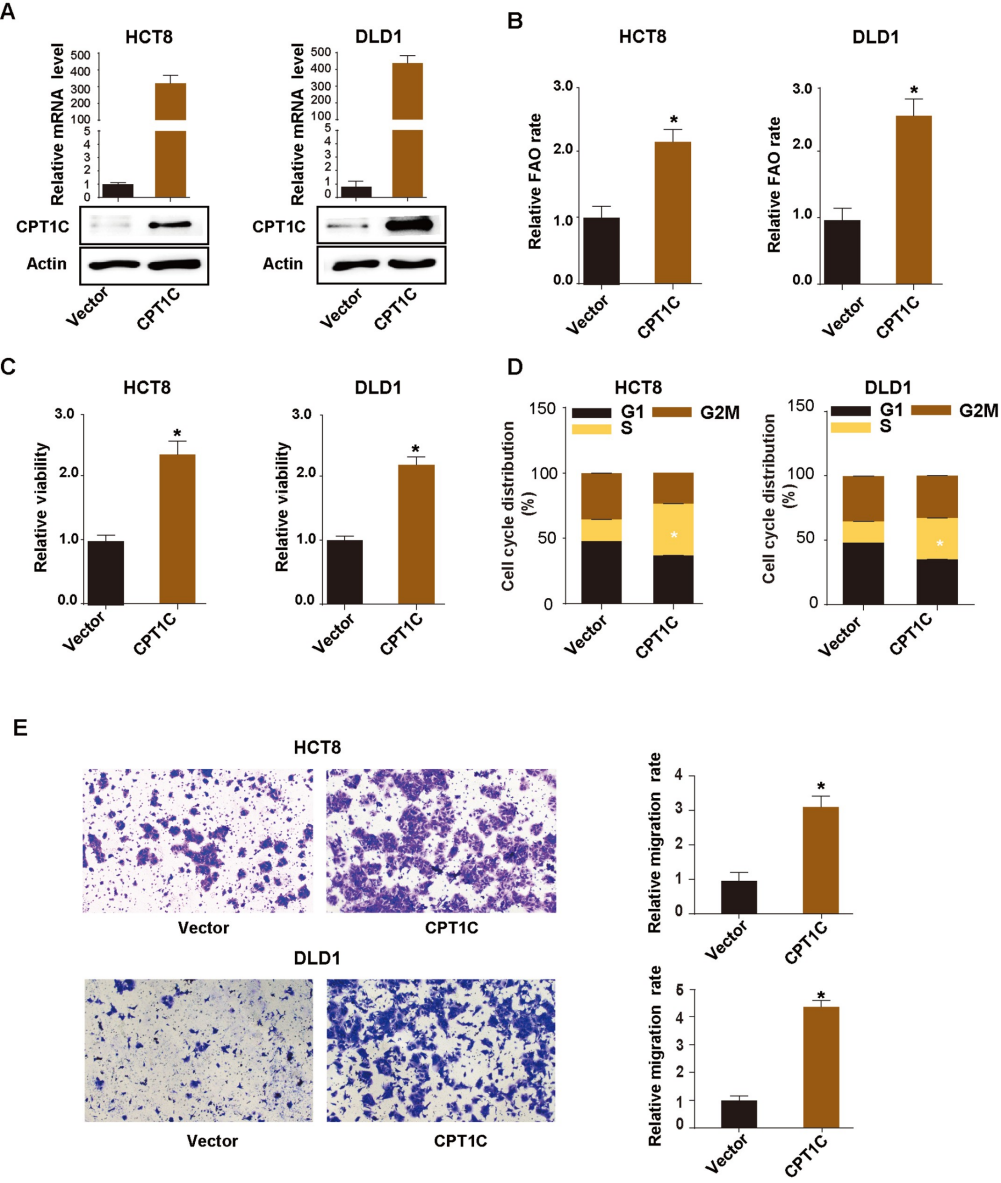
Figure 3 CPT1C overexpression promotes CRC cell proliferation and migration (A) qRT-PCR and western blot analysis showing enforced expression of CPT1C in CRC cells. (B,C) CCK-8 assay showing the viability of HCT8 (B) and DLD1 cells (C) at the indicated time points. (D) CPT1C increased the number of S-phase CRC cells. (E,F) CPT1C overexpression enhanced cell migration ability. Data are presented as the mean±SD. The P value was calculated using one-way ANOVA followed by Dunnett’s multiple comparisons. * P<0.05.

Basal CTP1C expression was detected in four CRC cell lines with distinct biological phenotypes, among which HCT116 and RKO cells showed relatively higher CPT1C expression ( Supplementary Figure S1B). To explore whether CPT1C could promote CRC cell growth and metastasis, we knocked down *CPT1C* with its specific small short hairpin RNA in HCT116 and RKO cells ( Figure 2A). Downregulation of CPT1C expression markedly inhibited the FAO rate ( Figure 2B), depressed cell proliferation ability ( Figure 2C), caused cell cycle arrest ( Figure 2D) and inhibited cell migration ( Figure 2E,F). To further investigate the effects of enforced CPT1C expression on cell proliferation, CPT1C was overexpressed in another two cell lines, HCT8 and DLD1 ( Figure 3A). In contrast to the cells transfected with vector, CRC cells with enforced CPT1C expression had an increased FAO rate ( Figure 3B), with facilitated cell proliferation ( Figure 3C), prolonged S phase ( Figure 3D) and enhanced cell migration ability ( Figure 3E,F). To unveil the molecules mediating cell proliferation and migration, we detected the expressions of proliferating cell nuclear antigen (PCNA), cyclin D1 and E-cadherin in cells with downregulated CPT1C expression. The results showed that attenuation of CPT1C expression decreased PCNA, cyclin D1 and E-cadherin expressions ( Supplementary Figure S1C), while overexpression of CPT1C increased PCNA, cyclin D1 and E-cadherin expressions ( Supplementary Figure S1D).

### FAO mediated by CPT1C is critical for cell growth and metastasis

**Figure 4:**
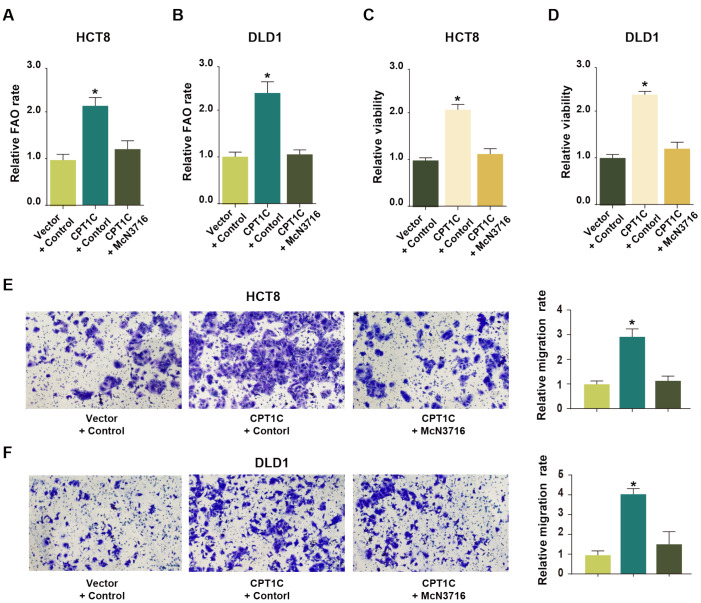
Figure 4 FAO mediated by CPT1C is required for CRC cell growth (A,B) McN3716 treatment notably inhibited the FAO rate in HCT8 (A) and DLD1 (B) cells with ectopic CPT1C expression. (C,D) McN3716 treatment notably inhibited cell viability in HCT8 (C) and DLD1 (D) cells with ectopic CPT1C expression. (E,F) McN3716 treatment notably suppressed cell migration in HCT8 (E) and DLD1 (F) cells with ectopic CPT1C expression. Data are presented as the mean±SD. The P value was calculated using one-way ANOVA followed by Dunnett’s multiple comparisons. * P<0.05.

To further probe whether CRT1C-regulated FAO is pivotal for CRC cell growth and metastasis *in vitro*, McN3716, an FAO inhibitor, was applied to treat CRC cells with CPT1C overexpression. The results demonstrated that the addition of McN3716 almost completely reversed the effects of CPT1C overexpression on the FAO rate ( Figure 4A,B), cell proliferation ( Figure 4C,D) and cell migration ability ( Figure 4E,F).

### CPT1C is transcriptionally activated by HIF1α

**Figure 5:**
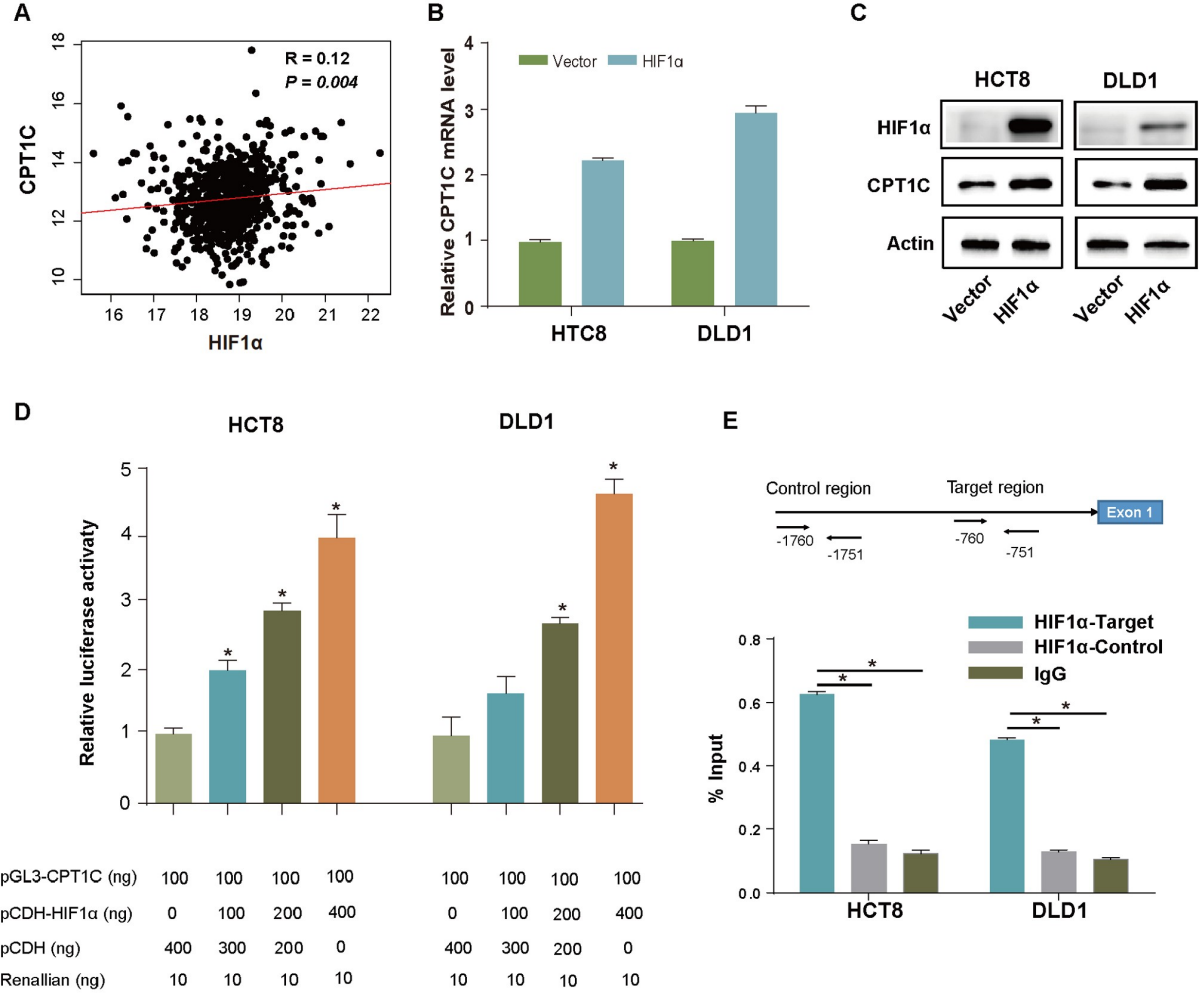
(A) Correlation analysis between CPT1C and HIF1α expressions in the TCGA database. (B,C) qRT-PCR and western blot analysis showing that HIF1α overexpression enhanced CPT1C expression in HCT8 (B) and DLD1 (C) cells. (D) Luciferase reporter assay showing that HIF1α transcriptionally activated CPT1C expression. (E) ChIP results in HCT8 and DLD1 cells showing that HIF1α can bind with the *CPT1C* promoter. Data are presented as the mean±SD. The *P* value was calculated using one-way ANOVA followed by Dunnett’s multiple comparisons. * *P*<0.05.

The hypoxic microenvironment plays pivotal roles in tumor progression, and the transcription factors of the hypoxia-inducible factor family have been established as important regulators of fatty acid metabolism-related genes. Moreover, hypoxia-inducible factor 1 alpha (HIF1α) is the most extensively studied member. Based on analysis of the TCGA cohort, we observed a positive correlation between the expression of CPT1C and HIF1α level ( *P*=0.004; Figure 5A). To further verify whether HIF1α could transcriptionally activate CPT1C, we overexpressed HIF1α in two CRC cell lines and confirmed that the mRNA and protein levels of CPT1C were significantly elevated upon HIF1α overexpression ( Figure 5B,C). Furthermore, luciferase reporter assay showed that HIF1α overexpression in CRC cells noticeably enhanced the luciferase activity of the *CPT1C* promoter, indicating that HIF1α could transcriptionally activate CPT1C ( Figure 5D).

To further confirm whether HIF1α binds directly to the promoter region of *CTP1C*, we used the JASPAR database to determine the potential binding sites and selected the binding site with the highest score (‒760~‒751: 5′-AGACGTGCCC-3′) for further experiments. ChIP-qPCR analysis indicated that HIF1α could bind to the promoter region of *CPT1C* directly ( Figure 5E).

## Discussion

Previous reports showed that the proliferation and migration of colon cells are provoked by the stimulation of a series of oncogenes [ 19- 21] . In this study, we unveiled for the first time the correlation between CPT1C expression level and metastatic potential in CRC. In addition, high expression of CPT1C is associated with poor RFS in CRC patients. Further *in vitro* experiments showed that CPT1C-mediated FAO is essential for expediting cell proliferation and enhancing cell migration in CRC cells. Intriguingly, we demonstrated that CPT1C expression is elevated in response to hypoxia and that HIF1α is a transcriptional target of HIF1α.

Metabolic reprogramming has been a hotspot in cancer research for decades, and dysregulation of lipid metabolism is one of the most crucial metabolic hallmarks of cancer cells. Furthermore, it is widely accepted that reprogramming of lipid metabolism occurs frequently in tumor development and progression [ 21– 24] . Consumption of lipids by FAO can provide energy for cancer cells for growth and metastasis. Under nutrient-depleted conditions, cancer cells upregulate specific enzymes catalyzing FAO to favor fatty acids as an energy source [ 25– 27] . Consequently, FAO-induced energy production could be of great significance for cancer cells to withstand metabolic stress, which prevails during tumor progression. Accumulating evidence has revealed the significance of FAO in maintaining the survival of cancer cells, including prostate cancer, ovarian cancer and breast cancer cells. Furthermore, inhibiting FAO by targeting certain enzymes could be a novel and effective approach to suppressing cell viability, indicating that FAO-related genes could be potential targets for drug development [ 28– 31] .

CPT1s, including CPT1A, CPT1B and CPT1C, are important rate-limiting enzymes involved in FAO. In this study, we explored their expression levels and prognostic values in CRC for predicting RFS based on the public TCGA database, which revealed that CPT1C is the only prognostic marker for RFS. However, the expression levels of CPT1A and CPT1B showed no association with disease relapse risk. The roles of CPT1C in cancer have scarcely been investigated, and few studies have attempted to investigate the prognostic value of CPT1C in CRC. To date, the notion that CPT1C could promote malignant phenotype transition has been revealed in several other cancers. In gastric cancer, FAO mediated by CPT1C was proven to be an important factor promoting cell proliferation [32]. In addition, Gao *et al*. [33] reported that CPT1C could promote the ovarian metastasis of gastric cancer. CPT1C was found to be a target of miR-1291, and ectopic CPT1C expression could promote cell proliferation [33]. Based on these previous reports, we speculated that CPT1C may play an important role in mediating CRC progression. Therefore, we further intervened in the expression of CPT1C in CRC cells and demonstrated that CPT1C could promote cell proliferation and migration by activating FAO. Previous research exploring the roles of CPT1s in other cancer types has revealed that CPT1s expression is positively associated with hypoxia, and CPT1A has been confirmed as the direct target of HIF1α [34]. In this study, we validated that CPT1C expression could be transcriptionally upregulated by HIF1α, emphasizing the role of HIF1α in reprogramming lipid metabolism in CRC.

Nevertheless, although we unveiled the prognostic and tumorigenic roles of CPT1C in CRC based on RNA-seq data from public database, it has not been externally validated in another cancer center. Additionally, immunohistochemical staining of CPT1C should be further performed to evaluate its prognostic value at the protein level. Moreover, although we mainly explored the roles of CPT1C in CRC from the perspective of prognosis prediction and biological functions, we could not exclude the possibility that CPT1A and CPT1B might also participate in tumor phenotype modulation because some previous studies have reported that CPT1A and CPT1B could promote cell invasion and proliferation in other malignancies [ 35, 36]

In conclusion, for the first time, we revealed that CPT1C is a prognostic marker in predicting relapse risk for CRC patients, while the enhanced FAO mediated by CPT1C is essential for maintaining CRC cell growth and enhanced migration ability, which makes CPT1C a potential therapeutic target.

## Supporting information

Figure_S1

## Supplementary Data

Supplementary data is available at *Acta Biochimica et Biophysica Sinica* online.
