# Supplementary figures and images for: CPT1C-mediated fatty acid oxidation facilitates colorectal cancer cell proliferation and metastasis: CPT1C promotes CRC progression
Source: Acta Biochim Biophys Sin (Shanghai). 2023 Apr 20;55(8):1301–9. doi: 10.3724/abbs.2023041 (PMC10448059; doi:10.3724/abbs.2023041)

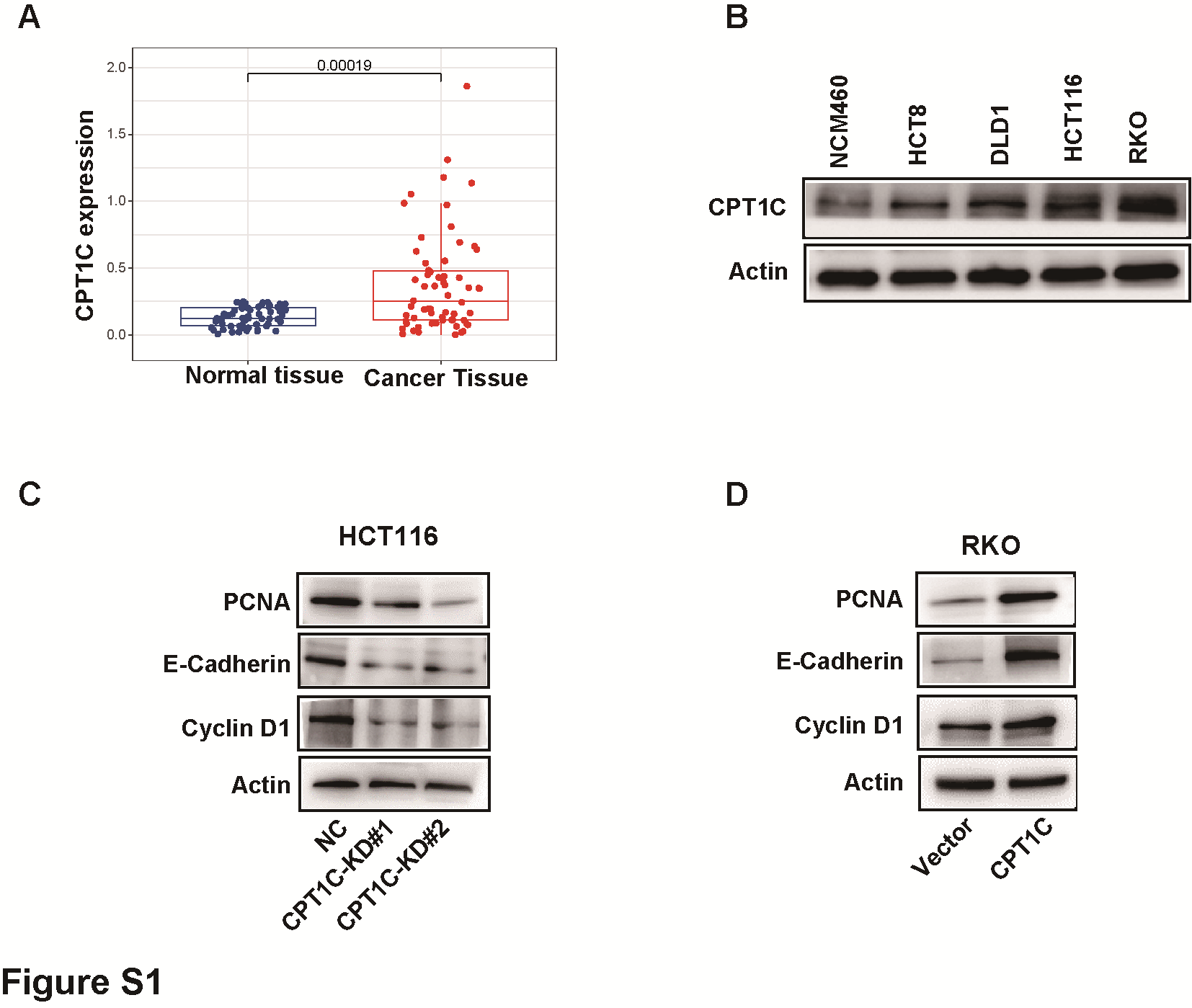

Supplement: Figure_S1 [file Figure_S1.png]
